# Pulmonary benign metastasizing leiomyomas: a case series of 23 patients at a single facility

**DOI:** 10.1186/s12890-020-01330-4

**Published:** 2020-11-10

**Authors:** Rong Fan, Fengzhi Feng, Hua Yang, Kaifeng Xu, Shanqing Li, Yan You, Xirun Wan, Lan Zhu

**Affiliations:** 1 Department of Obstetrics & Gynecology, Peking Union Medical College Hospital (PUMCH), Chinese Academy of Medical Sciences and Peking Union Medical College (CAMS & PUMC), No. 1, Shuai Fu Yuan, Dong Cheng District, Beijing, China; 2 Department of Pulmonary and Critical Care Medicine, PUMCH, CAMS & PUMC, Beijing, China; 3 Department of Thoracic Surgery, PUMCH, CAMS & PUMC, Beijing, China; 4 Department of Pathology, PUMCH, CAMS & PUMC, Beijing, China

**Keywords:** Pulmonary benign metastasizing Leiomyomas, Follow-up, Outcomes

## Abstract

**Background:**

Pulmonary benign metastasizing leiomyoma (PBML) is a rare disease characterized by leiomyoma of benign histopathology existing in the lungs. Because of its rarity, limited literature with a single case or small number of cases has been regarding to the clinical course, pathology or management of PBML.

**Methods:**

A retrospective study was performed of all PBML cases diagnosed and managed at Peking Union Medical College Hospital (PUMCH) from 2001 to 2019. The clinical characteristics, pathology, treatment and outcomes of each case were studied.

**Results:**

There were 25 PBML patients identified in the 19-year period in PUMCH, and 23 patients’ data was analyzed. The median age at diagnosis was 46 years. There were 7 patients (30.4%) diagnosed with postmenopausal status. Two patients (8.7%) had no uterine leiomyoma, and 3 patients (13.0%) had no gynecologic surgery history. Immunohistochemistry of most lesions demonstrated positive for desmin, SMA and Estrogen/Progesterone Receptors; and negative for S-100 were shown in 7 cases. After curative or diagnostic surgeries for the PBML, several treatments from observation to medical or surgical castration were performed. Nine premenopausal patients preserved their ovaries at first. At a median follow-up of 8 years, 3 patients finally had oophorectomy.

**Conclusions:**

PBML is a rare disease and should be treated by individualization according to the patients’ age, symptoms and extent of lesion. Curative surgery for patients with limited lesions can achieve the complete response. For patients that are young and asymptomatic, close observation is recommended as the first choice. All patients should undergo long-term surveillance.

## Background

Benign metastasizing leiomyoma (BML) is a rare disease that is characterized by lesions made of benign-looking aggregates of smooth muscle cells located in any site of the body, such as lung, abdominal cavity, retroperitoneum, muscular tissue, lymph node, blood vessels, or heart [1]. Lungs are the most common metastatic site, most pulmonary BML (PBML) are discovered mainly in premenopausal women and are characterized by the presence of multiple, bilateral lung nodules of different sizes that may be mistaken for metastatic cancer. A definitive diagnosis requires a medical history, imaging studies, and pathologic examination of tumors [1, 2]. There is no standardized treatment for BML due to its rarity. Careful observation, surgical resection of metastatic lesions, oophorectomy or antiestrogen therapy has been published [3–5].

Because of the rarity of PBML, literature regarding this tumor mostly is a single case or cases reports [3–5]. To the best of our knowledge, there are only two studies in the literature contained a relatively large 10 cases of PBML to date [2, 6]. Furthermore, there was seldom article thoroughly investigating the outcomes of patients with PBML, especially in young women desired to preserve ovarian function. Our study seeks to further describe the clinical characteristics of PBML and to propose recommendations for the treatment and follow-up of these patients.

## Methods

The Institutional Review Board of Peking Union Medical College Hospital (PUMCH) approved this retrospective study. We reviewed cases diagnosed primarily or in consultation at PUMCH with pathological diagnosis of PBML from Jan. 2001 to Sep. 2019. Eligible patients were identified using a medical records database. Lung nodules either achieved by biopsy or surgical resections were histologically defined as a PBML with (1) the presence of benign proliferative smooth muscle cells in intersecting fascicles, (2) low cellularity, (3) low mitotic index, (4) the absence of nuclear atypia and tumor necrosis, (5) no invasion to surrounding tissue, and/or (6) at immunohistochemistry, positive staining for Desmin, Smooth Muscle Actin (SMA), estrogen receptor (ER) and progesterone receptor (PR), and/or negative for CD117, CD34, and S-100. The same pathologist (Dr. Yan You) in our institution reviewed their histopathological slides. Exclusion criteria were male patients, or without histological confirmation.

The medical records of eligible patients were reviewed and data were collated that included their clinical characteristics, age at diagnosis, surgical procedure for diagnosis, imaging findings, other sites of metastatic disease, gynecologic history for uterine leiomyomas, treatment options, and outcomes of follow-up. According to the medical records, patients’ follow-up included a clinical examination, chest CT, and pelvic ultrasound examination.

PBML response to therapy was evaluated by tumor burden on axial imaging planes from CT employing RECIST 1.1 guidelines [7]. The PBML lesions (up to a maximum of 2 lesions total) were recorded in at least one dimension [8]. Complete response (CR) was defined as the disappearance of all target lesions, partial response (PR) was defined as at least a 30% decrease in the sum of the longest diameter of target lesions, progressive disease (PD) was defined as at least a 20% increase in the sum of the longest diameter of target lesions, and stable disease (SD) was defined as neither sufficient shrinkage to qualify for partial response nor sufficient increase to qualify for progressive disease [7].

Descriptive statistics regarding patients’ characteristics were calculated. Demographic and baseline characteristics were summarized by using medians (ranges) for continuous variables and proportions for categorical variables. The categorical variables were tested by Fisher’s exact test. Statistical significance was determined by a *p* value of < 0.05. Statistical analysis was performed using commercially available software (SPSS version 23.0, IBM Corp, Armonk, NY).

## Results

Over the 19-year study period, 25 patients were primarily diagnosed and treated at or referred to our facility for PBML. Two patients were excluded due to male patient (*n* = 1), or slides were not available for review (*n* = 1). Five patients were followed up no more than 6 months, their baseline characteristics were described and analyzed, but their follow-up data were not included.

The clinical characteristics Of the 23 patients are shown in Table 1. The median age at diagnosis was 46 years (range, 36–62 years). Seven patients (30.4%) were identified as postmenopausal. Nineteen patients (82.6%) had a history of uterine leiomyoma; 2 patients (8.7%) had no history of uterine leiomyoma and 2 patients (8.7%) did not exam for it. Of 19 patients with history of uterine leiomyoma, 16 patients (84.2%) had undergone at least one surgical procedure for uterine leiomyoma prior to diagnosis of PBML, with the median time from the first gynecologic surgery of 10.5 years (range, 2–15 years), and 3 patients had no surgical history. Thirteen patients (56.2%) were identified with pulmonary tumors due to their respiratory or other symptoms such as cough (*n* = 7), short of breath (*n* = 3), chest pain (*n* = 1), backache (n = 1) and bloating (n = 1). The other 10 patients (43.8%) were incidentally detected by chest imaging procedures done for other reasons such as health care examination (*n* = 9) or follow-up after thyroid cancer (*n* = 1).

**Table 1:** Clinical details of the 23 pulmonary benign metastasizing leiomyomas in Peking Union Medical College Hospital series

Case No.	Time to Diagnosis From Gynecologic Surgery (y)	Symptoms	Radiographic Findings	Surgical Procedure for Diagnosis	Immunohistochemistry	Follow up
1	N/A	Cough	Right lower lobe nodule, single	Thoracotomy, resection of the right middle segment of the lung	SMA(+), Desmin(+), S-100(−)	14 year, CR
2	N/A	Cough	Right pulmonary hilum nodule, single	Thoracotomy, right upper lobe resection	N/A	18 years, CR
3	2	No	Right upper lobe multiple nodules	Thoracotomy, wedge resection of right upper lobe	N/A	17 years, SD
4	11	No	B/L multiple nodules	VATS, wedge resection	SMA(+), Desmin(+),ER(+/−), PR(+), S-100(−)	11 years, SD
5	N/A	Cough	Left main bronchus nodule, single	Thoracotomy, resection of left lung	SMA(+), Desmin(+), S-100(−), Ki-67 < 1%	8 years, CR
6	14	Chest pain	B/L multiple nodules	VATS, wedge resection of left lower lobe	ER(++), PR(++), Ki-67 (1%)	8 years, SD
7	N/A	No	Left upper lobe nodule, single	VATS, resection of left upper lobe	SMA(++), Desmin(+++), S-100(−)	9.5 years, CR
8	11	Short of breath	B/L multiple nodules	Biopsy	CD34(−), CD117(−), Desmin(+), SMA(+), Ki-67(1%), S-100(−)	3 years, PD
9	10	Short of breath	B/L multiple nodules	VATS, wedge resection of left lower lobe	ER(+), PR(+),Desmin(+), SMA(+), Ki-67 (3%)	1 year, SD
10	7	No	B/L multiple nodules	Biopsy	ER(+), PR(+), KI-67 < 1%	9 years, SD
11	10	Cough	B/L multiple nodules	Biopsy	SMA(+), ER(+), PR(+), Desmin(+), Ki-67(3%), CD34(−), S-100(−)	1.5 year, SD
12	2	Short of breath	B/L multiple cysts, pneumothorax	VATS, wedge resection of left upper lobe	ER(+), PR(+),Desmin(+), Vimentin(+)	1.5 year, SD
13	14	No	B/L multiple nodules	Biopsy	N/A	17 months, PR
14	6	No	B/L multiple nodules	Biopsy	N/A	4 years, PD
15	9	No	B/L multiple nodules	Biopsy	SMA(+), Desmin(+),BCL-2(+),Ki-67 (3%)	2 years, PR
16	N/A	Cough	B/L multiple nodules	VATS, right upper lobe resection	N/A	19 months, SD
17	5	No	B/L multiple nodules	VATS, resection of left pulmonary mass	ER(+++), PR(+++), Desmin(+++), Ki-67<1%	11 year, PR
18	14	Backache	B/L multiple nodules	Biopsy	SMA(+), Desmin(+), Caldesmon(+), Ki-67 < 1%	2 months, not included
19	N/A	Bloating	B/L multiple nodules	Biopsy	N/A	6 months, not included
20	N/A	No	B/L multiple nodules	Biopsy	SMA(+), Desmin(+), ER(+), PR(+), Ki-67 (2%)	12 years, SD
21	12	No	B/L multiple nodules	Biopsy	ER(+),PR(+),SMA(+),S-100(−)	6 months, not included
22	14	Cough	B/L multiple nodules	VATS	SMA(+),Desmin(+), ER(+), PR(+)	2 months, not included
23	15	Cough	B/L multiple nodules	Biopsy	SMA(+),Desmin(+), ER(++), PR(+), Ki-67(1%)	4 months, not included

**Fig. 1:**
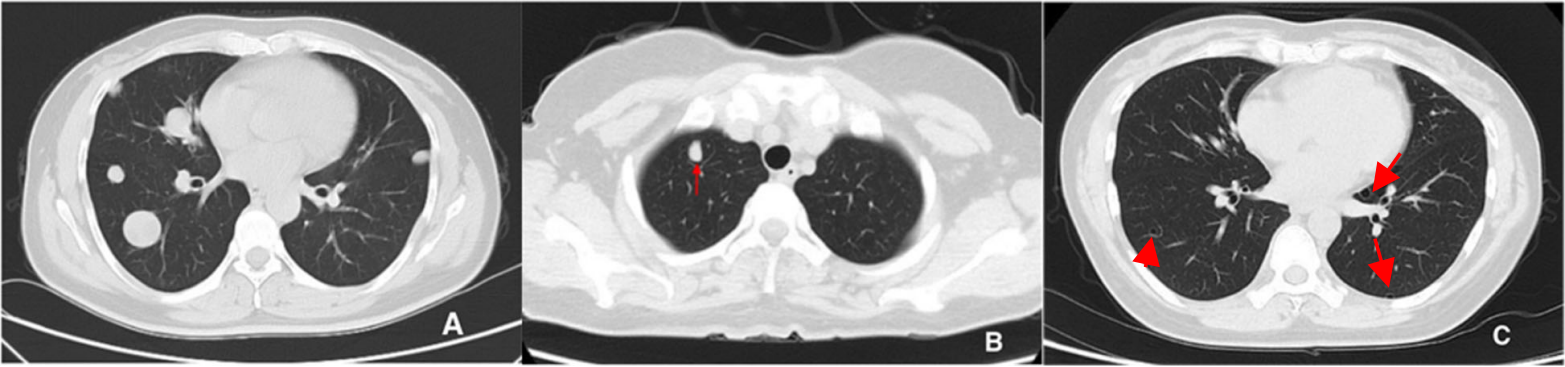
Chest computed tomography (CT) showed pulmonary benign metastasizing leiomyoma. **a** bilateral multiple nodules. **b** single nodule (arrow). **c**: bilateral multiple cysts (arrows)

**Fig. 2:**
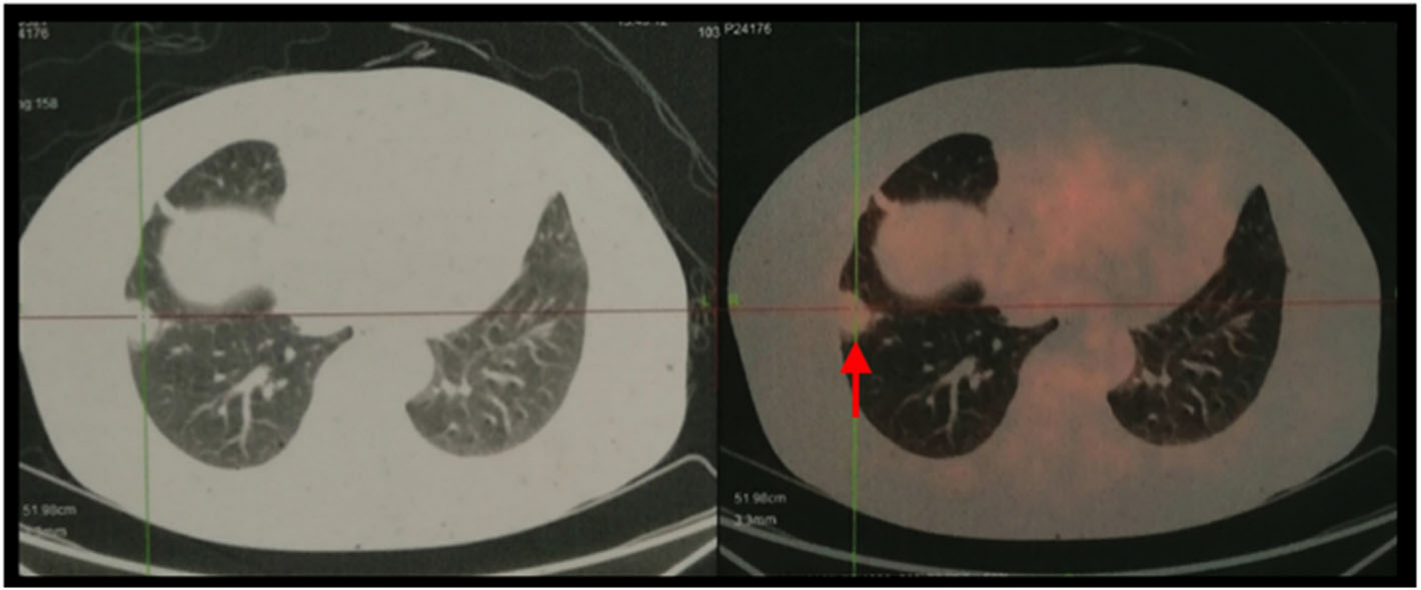
PET-CT showed mild uptake of 18F-FDG (SUVmax 2.1, arrow)

All lesions were identified by chest CT. Multiple pulmonary nodules were identified in 18 patients (78.3%), of which 17 patients with bilateral diseases (Fig. 1a, patient 13) and 1 with unilateral diseases. There were 4 patients (17.4%) with solitary PBML nodules (Fig. 1b, patient 7), and 3 of them were postmenopausal. There was 1 patient had multiple cystic diseases (Fig. 1c, patient 12). In addition, PET-CT scan was performed in 11 patients (47.8%) and showed no uptake or only mild uptake of 18F-FDG (Fig. 2, patient 23), with a median SUV of 2.0 (range, 1.5–3.8). Clinical physical examination and radiologic images revealed apparent extrapulmonary diseases in 3 patients (13.0%) including disseminated peritoneal leiomyomatosis (*n* = 1), pelvic retroperitoneum mass (n = 1), and pelvic retroperitoneum mass accompanied with left medial thigh muscle mass (*n* = 1).

All 23 patients had undergone surgical procedures of pulmonary nodules for diagnosis including biopsy, wedge resection and lobectomy. Four patients underwent thoracotomy, 8 patients underwent video-assisted thoracoscopic surgery (VATS), and 11 patients were diagnosed by Computed tomography–guided biopsy. One patient with left medial thigh muscle mass simultaneously underwent biopsy of both lung and left thigh muscle. Pathologic findings in all tissue samples met diagnostic criteria of BML. Four patients (17.4%) with solitary PBML nodules underwent a curative surgical resection (3 patients with wedge resection and 1 patient with left pneumonectomy because the mass was in the left main bronchus). The historic and operative characteristics are provided in Table 2.

**Table 2:** Historic and operative characteristics of 23 PBML cases

Variables	n (%)
Menopause	
Yes	7 (30.4%)
No	11 (47.8%)
Unknown	5 (21.7%)
History of uterine leiomyoma	
Yes	19 (82.6%)
No	2 (8.7%)
Unknown	2 (8.7%)
Prior gynecologic surgery procedures for uterine leiomyoma	
No	7 (30.4%)
1	10 (43.5%)
2	5 (21.7%)
3	1 (4.3%)
Gynecologic procedure before diagnosis of PBML	
Myomectomy	7 (43.75%)
Hysterectomy	9 (56.25%)

Additional treatment strategy including close observation, surgical castration, medical castration with gonadotropin-releasing hormone analogs (GnRHa), estrogen receptor antagonists (tamoxifen), or aromatase inhibitors (letrozole). After surgical resection of PBML, 14 patients considered observation, 5 patients underwent bilateral salpingo-oophorectomy, and 4 patients selected adjuvant medicine treatment. Of 14 patients with observation, only one patient (patient 10) had hysterectomy and bilateral salpingo-oophorectomy because of symptomatic uterine leiomyoma and progression of PBML after 8 years. All Of 5 patients with bilateral salpingo-oophorectomy had a close observation and no disease progression. Of 4 patients with adjuvant medicine treatment, 3 patients received GnRHa injections (3.75 mg/month) and 1 patient took letrozole (2.5 mg/day). 2 of 3 patients with GnRHa finally had bilateral salpingo-oophorectomy because their pulmonary nodules shrinked after injections but grew again after stop the medicine. Patient 17 was the most special one; she had 6 doses of GnRHa injections after diagnosis of PBML. Shortly after stopping GnRHa, the size of PBML grew slowly again, and then she underwent laparoscopic bilateral salpingo-oophorectomy. The tumor shrinked subsequently. However, bilateral pulmonary nodules on radiographic surveillance were enlarged again 3 years after surgical castration. Since then, she has been taking letrozole 2.5 mg oral daily and achieved again PR 6 months after medical treatment. To the date of writing the manuscript, she has still been under treatment.

Because 5 patients were followed up no more than 6 months, their follow-up data was not included. At a median follow-up of 8 years (range, 1–18 years), 4 of 18 (22.2%) patients achieved CR, 3 (16.7%) patients achieved PR, 9 (50%) patients had SD, and 2 (11.1%) patients had PD. The clinical characteristics between patients achieved CR + PR and SD + PD were compared in Table 3. Only the characteristics of pulmonary lesions showed a significant difference between the two groups. For the 4 patients with CR (median follow-up: 11.75 years), all of them had single nodule in lung and received curative surgical resection without additional therapy. For the 3 patients achieved PR (median follow-up: 2 years), all of them received surgical castration. Of 9 patients with SD, 6 patients had only a careful observation with a median follow-up for 1.5 years (range: 1–17 years), 2 patients underwent surgical castration (8 and 11 years of follow-up), and 1 patient took letrozole for 1 year. For the 2 patients with PD, patient 14 was young and preserved her ovaries. She was followed up for 4 years, her lesion was stable at the first 3 years and grew in the past year, she chose to continue close observation because she had no symptom; patient 8 had 9 injections of GnRHa, her pelvic and pulmonary lesions grew after stopping the medicine, then she underwent bilateral salpingo-oophorectomy and surgical excision of pelvic retroperitoneum mass. She is now 6 months after the last surgery; the nodules in her lungs are stable for now.

**Table 3:** Clinical Characteristics between patients achieved CR + PR and SD + PD

	CR + PR	SD + PD	*P* value
Age (year)	47 (44 ~ 54)	44 (36–62)	0.635
Gynecologic surgery history (n)			
Yes	3 (42.9%)	9 (81.8%)	0.141
No	4 (57.1%)	2 (18.2%)	
Menopause			
Yes	4 (66.7%)	3 (33.3%)	0.315
No	2 (33.3%)	6 (66.7%)	
Pulmonary lesion characteristic (n)			
Single	4 (57.1%)	0	0.011
Multiple	3 (42.9%)	11 (100%)	
Diagnostic procedure (n)			
Surgery	5 (71.4%)	6 (54.5%)	0.637
Biopsy	2 (28.6%)	5 (45.5%)	

*CR* complete response, *PR* partial response, *SD* stable disease, *PD* progressive disease

Interestingly enough, after being diagnosed of PBML, 9 premenopausal patients preserved their ovaries at first, with the median age of 43 years (range, 36-51 years). Among them, 6 patients had close observation and 3 received GnRHa injections. Finally, only 3 patients (patient 8、10 and 17) underwent bilateral salpingo-oophorectomy during 1 year, 2.5 years and 8 years of follow-up, respectively.

## Discussion

This study represents the PUMCH experience over the past 19 years of diagnosing and managing women with PBML. Benign metastasizing leiomyoma was first recognized by Steiner in 1939 [9], when it was named as fibroleiomyomatous hamartoma. PBML is rare, therefore, most of the articles about it are case reports [3–5, 10–13]. To the best of our knowledge, this is the largest series of patients with PBML in a single facility. It is also the second to address long-term follow-up in patients with PBML [6]. The median age at diagnosis of our study is 46 years (range, 36–62 years). In another systematic review of BML, the average age of the patients is 47.3 years [14]. Almost 70% of the patients suffered from myomectomy or hysterectomy because of uterine leiomyoma before diagnosis of PBML, the percentage in the other two studies are 80 and 100% [2, 6]. 26% of the patients had twice and above prior gynecologic procedures. Symptoms are not specific, and almost half of the patients are asymptomatic. The CT images of PBML are not unique, the majority of the patients present with multiple solid nodules (78.2%). In those cases that are hard to differentiate, 18F-FDG-PET has helped to distinguish PBML from malignant diseases such as leiomyosarcoma or other metastasis cancers, because PBML showed much lower uptake. According to previous studies and our result, the possibility of PBML should be considered in post-leiomyoma treatment patient populations with abnormal chest CT images of single or multiple nodules.

The definite diagnosis was depending on pathology. For diagnostic purposes, immunohistochemistry of some lesions with antibodies against desmin, smooth muscle actin, vimentin, and Caldesmon are positive, which confirm mesenchymal derivation with smooth muscle differentiation of these tumors. The majority of the lesions displayed an expression of hormone (estrogen and progesterone) receptors with strong intensity, which provides us therapeutic strategies. The Ki-67 index of the tumor was low (from < 1 to 3%), which showed indolent characteristic. The clinical course of BML also reflects a slow process. S-100 is an antigen that may be used to distinguish BML from other smooth muscle tumors, which appears to be expressed at low levels in BML [1]. In our series, there were 7 cases with S-100 staining, which all expressed negative.

So far, several hypotheses on the histogenesis of these lesions have been suggested. The most accepted hypothesis is that cells of pulmonary BML nodules are derived from uterine cells dislodged from the uterus at the time of myomectomy or hysterectomy performed for treatment of uterine leiomyoma [1, 15]. It is interesting that there are 2 patients in our case series did not have a history of leiomyoma. These 2 patients are both postmenopausal and with a single nodule. And there are 3 patients diagnosed of uterine leiomyoma before or at the same time of PBML diagnosis, but without surgery treatment; 2 of 3 are postmenopausal. According to a systematic review, there are 10 cases of women diagnosed of BML who had not undergone prior surgery [14]. Therefore, these cases may suggest different histogenesis of BML. It is also possible that BML could originate from lung smooth muscle cells or other sites containing smooth muscle cells. Whole genome sequencing data from BML samples and leiomyoma samples may be required to accurately estimate origin of BML per case in future studies. Unfortunately, collecting these samples remains a major challenge, owing to the difficulties of collecting multiple tissue samples at different diagnosis time points and the costs of genomic profiling.

BML is most commonly found in premenopausal women. The formal researches considered the BML’s growth are dependent on hormone stimulation. This view of point was supported by the BML size reduction that has been observed and reported in menopause, after termination of pregnancy, and in cases of surgical or chemical castration [5, 16–18]. In our case series, the two patients with progressive disease are both young and preserved their ovaries, and patient 8’s lesion was temporarily stable after oophorectomy. These results suggest us that estrogen may be the main cause of disease progression. Therefore, we suggest that anti-estrogen therapy should be considered for these patients. However there are 7 postmenopausal patients in our study. Postmenopausal patients also been reported in other studies [6, 16], though they were a small part of all the PBML patients. In another series of older patients, the author also reported 2 of the 3 postmenopausal cases with hormonal manipulation demonstrated disease progression [6]. One case in our study also had minimal growth of PBML even if receiving GnRHa treatment. These cases suggest us there may be some other biologic controls of PBML other than hormone, especially in postmenopausal patients.

Because of the limited understanding of BML, standard treatment has still not been established. Our study results showed that for patients with single or limited nodules, a curative surgery for the removal of pulmonary lesions is important for achieving a good outcome. In the past, some premenopausal patients received surgical castration for the first choice of treatment to BML [13, 17]. The side effect of estrogen deficiency includes vasomotor symptoms, osteoporosis, cardiovascular disease, cognitive decline and dementia, which will negatively affect their quality of life, even associated with increases in morbidity and mortality [19]. In our series, two thirds of premenopausal patients with preserving ovaries had good prognosis. Besides, PBML of patient 17 even grew after the surgical castration. So the role of oophorectomy for patients with BML is still unknown. Therefore, for young women with PBML, especially those with asymptomatic multiple nodules, ovary-sparing therapy and close monitoring is recommended.

Although the present study was retrospective design and the small number of study subjects, and the optimal treatment options remained to be defined, these limitations were unavoidable due to the rarity of BML. However, our series encountered many special clinical features of PBML, including postmenopausal women, no history of uterine leiomyoma, coexistence of BML in other sites, multiple cystic vacuolar nodules on chest CT morphologically mimicking lymphangioleiomyomatosis, and shrinking and enlarging again of pulmonary multiple nodules after castration surgery and so on. All of these clinical characteristics may provide important reference value for diagnosis and treatment of PBML.

## Conclusions

PBML is a rare disease that commonly found in premenopausal women. However, it can also be found in postmenopausal women. An individual treatment strategy should be considered for each patient depending on the size and location of the tumor and the hormone status. In patients with limited nodules, a curative surgery to resect the pulmonary lesion is important. This disease progression is indolent. In young asymptomatic women, if the nodules are not resectable, we’d better first choose conservative treatment instead of surgical castration. Disease progression and complications may occur after many years. All patients should undergo long-term surveillance by computed tomography scan.

## Data Availability

The datasets used and analysed during the current study are available from the corresponding author on reasonable request.

## Abbreviations

PBML: pulmonary benign metastasizing leiomyoma
SMA: Smooth Muscle Actin
ER: estrogen receptor
PR: progesterone receptor
CR: complete response
PR: partial response
PD: progressive disease
SD: stable disease
VATS: video-assisted thoracoscopic surgery
GnRHa: gonadotropin-releasing hormone analogs.
